# Deep Learning‐Based OCT Segmentation for Stiffness Quantification in Evaluating Low‐Level Laser Therapy for Wound Healing

**DOI:** 10.1002/jbio.70291

**Published:** 2026-06-07

**Authors:** Gilang Titah Ramadhan, Yih‐Kuen Jan, Ben‐Yi Liau, Hsu‐Tang Cheng, Wei‐Cheng Shen, Chien‐Cheng Tai, Sheena Christabel Pravin, Kiruthika Venkataramani, Winson Chiu‐Chun Lee, Congo Tak Shing Ching, Chi‐Wen Lung

**Affiliations:** ^1^ Department of Informatics Tiga Serangkai University Surakarta Indonesia; ^2^ Rehabilitation Engineering Lab, Department of Health and Kinesiology University of Illinois at Urbana‐Champaign Urbana Illinois USA; ^3^ Department of Automatic Control Engineering Feng Chia University Taichung Taiwan; ^4^ Division of Plastic and Reconstructive Surgery, Department of Surgery Asia University Hospital, Asia University College of Medical and Health Science Taichung Taiwan; ^5^ Department of Food Nutrition and Health Biotechnology Asia University Taichung Taiwan; ^6^ Department of Creative Product Design Asia University Taichung Taiwan; ^7^ School of Public Health, College of Public Health, Taipei Medical University Taipei Taiwan; ^8^ School of Electronics Engineering, Vellore Institute of Technology Chennai India; ^9^ School of Mechanical, Materials, Mechatronics and Biomedical Engineering, University of Wollongong Wollongong Australia; ^10^ Graduate Institute of Biomedical Engineering, National Chung Hsing University Taichung Taiwan

**Keywords:** low‐level laser therapy, skin injury, soft tissue stiffness, wound healing

## Abstract

**Background:**

This study evaluated the short‐term biomechanical response of wound tissue following low‐level laser therapy (LLLT) by examining changes in skin stiffness, a surrogate biomechanical indicator of short‐term tissue response, across different limb regions.

**Methods:**

A 660 nm LLLT protocol was applied to wound sites. Skin stiffness was quantified using optical coherence tomography (OCT) combined with an air‐jet indentation system, enabling non‐contact measurement of tissue deformation. For accurate layer‐specific assessment, a U‐Net–based model was employed to automate OCT image segmentation.

**Results:**

The automated segmentation by the U‐Net model achieved a segmentation accuracy of 92%, facilitated precise segmentation of skin layers. LLLT significantly reduced skin stiffness after treatment, indicating an acute modulation of tissue compliance.

**Conclusion:**

Short‐duration LLLT reduces skin stiffness immediately post‐treatment, indicating its potential as a non‐invasive intervention to modulate the biomechanical environment of wounds.

**Trial Registration:** ClinicalTrials.gov identifier: NCT07177274

## Graphical abstract

The purpose of this study is to evaluate the short‐term biomechanical response of wound tissue following low‐level laser therapy (LLLT) by measuring changes in skin stiffness in different regions of the limb following LLLT. Following short‐duration LLLT, skin stiffness is significantly reduced. The integrated optical coherence tomography (OCT) and deep learning framework allows non‐invasive quantification of relative mechanical changes during wound healing, providing a promising approach to monitor biomechanical dynamics.

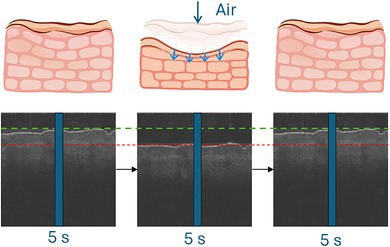

## 1 Introduction

Wounds are disruptions in the structural integrity of the skin and underlying tissues, which play a vital role in protecting the body against environmental factors [1]. Skin wounds, including acute injuries, are a significant public health issue worldwide, often requiring expensive treatments [2]. In 2022, the estimated expenditure on wound care in the United States reached $148.65 billion, underscoring the substantial financial burden associated with medical treatment and patient recovery [3]. In the future, the demand for wound treatment will grow significantly [4]. Untreated or poorly managed wounds can lead to complications such as infections, chronic pain, and systemic diseases, such as sepsis, which may have long‐term negative impacts on health and quality of life.

Effective treatment and prevention strategies are crucial for mitigating these effects and reducing the overall economic and health burdens [5]. Hyperbaric oxygen therapy and negative pressure wound therapy (NPWT) were previously considered advanced treatments that accelerated wound healing. HBOT delivers 100% oxygen under increased atmospheric pressure, promoting angiogenesis, increasing collagen production, and improving immune cell function [6]. NPWT supports healing by applying localized negative pressure to the wound, which removes excess fluid, reduces edema, encourages tissue granulation, and draws the wound edges together [7]. In recent years, low‐level laser therapy (LLLT) has gained popularity due to its non‐invasive nature, minimal side effects, and effectiveness in reducing inflammation, alleviating pain, and supporting tissue regeneration by stimulating mitochondrial activity and increasing adenosine triphosphate (ATP) production [8].

LLLT has been shown to modulate the skin environment to support wound healing by promoting cell proliferation, collagen synthesis, and microcirculation [9]. LLLT has shown promise in its biostimulatory effects. LLLT involves the use of specific light wavelengths to stimulate cellular activities, leading to improved collagen synthesis and facilitating wound healing in the dermis of the skin [10]. By delivering specific wavelengths of light, LLLT stimulates mitochondrial activity, leading to increased ATP production, which fuels the cellular processes essential for tissue repair [11]. This photobiomodulation effect enhances fibroblast proliferation and promotes collagen synthesis, particularly of type I collagen, which is crucial for restoring skin integrity. After therapy, the skin shows reduced inflammation, enhanced epithelialization, and more organized collagen deposition, indicating an improved tissue environment conducive to healing [12]. An increase in collagen synthesis, mainly type I collagen, not only supports the structural aspects of wound closure but also contributes to changes in skin stiffness, which is essential for restoring the skin's mechanical strength and resilience after injury [13].

Previous studies have utilized various metrics to quantify these properties, including Young's Modulus for elasticity and viscoelastic parameters [14, 15]. Other research has employed shear wave speed in elastography or indentation depth to characterize tissue resistance [16, 17]. While Young's Modulus represents a fundamental material property, its calculation often assumes tissue homogeneity and semi‐infinite geometry, which are rarely present in complex wound environments. In contrast, this study utilizes the structural stiffness coefficient as the primary biomechanical marker. This approach is preferred in clinical wound assessment because it captures the integrated response of the skin's architectural changes, including surface irregularities and layer‐specific alterations, providing a more pragmatic indicator of the tissue's functional state under LLLT [18]. Skin stiffness refers to the skin's resistance to deformation, which is influenced by the collagen and elastin network within the dermal layer. During wound healing, reduced stiffness is often indicative of edema or inflammation. In contrast, increased stiffness may indicate fibrosis or scar tissue formation, which is less ideal for maintaining the natural elasticity and function of the skin [19]. Assessing these parameters can provide critical insights into the acute biomechanical response, serving as a surrogate imaging marker of the acute tissue response to LLLT.

Wound healing is a complex and dynamic process that varies by anatomical location, with the upper and lower limbs exhibiting distinct recovery patterns due to differences in vascularization, mechanical stress, and tissue composition. Wound healing in the upper limbs is often accelerated because of the rich vascular supply and enhanced mobility, which facilitate efficient nutrient delivery and immune cell recruitment [20]. Additionally, the relatively more compliant skin in these areas may support more effective tissue remodeling and repairs. However, frequent movement at joint sites, such as the elbows and wrists, can paradoxically hinder healing by imposing repetitive tension on wound edges, potentially disrupting the repair process [21]. Wound healing in the lower limbs, particularly in the feet and ankles, is often delayed because of compromised blood circulation. Reduced perfusion in these locations is commonly associated with factors such as increased vascular resistance, peripheral arterial disease, venous insufficiency, and greater distance from the heart, which limits the efficient delivery of oxygen and nutrients [22].

Inadequate circulation impairs essential tissue repair processes, including angiogenesis, collagen deposition, and cellular migration, thereby prolonging healing time and increasing the risk of chronic wounds. In addition, the skin in this location tends to exhibit greater stiffness, driven by increased collagen cross‐linking and mechanical adaptations [23]. This altered biomechanical environment can reduce cellular responsiveness to external stimuli, such as photobiomodulation. As LLLT relies on light absorption by cells to trigger signaling pathways that promote proliferation and migration, excessive tissue stiffness may limit light penetration and impair downstream mechanotransduction, thereby potentially modulating the immediate response to LLLT and supporting wound healing. These contrasting characteristics between the upper and lower limbs underscore the critical role of regional vascularity, tissue compliance, and biomechanical properties in modulating the wound healing trajectory, highlighting the need for site‐specific assessment and tailored therapeutic strategies.

To accurately quantify these biomechanical variations, integrating image segmentation for tools to measure soft‐tissue biomechanics is essential. Manual assessment of optical coherence tomography (OCT) images is highly susceptible to localized tissue heterogeneity and surface irregularities inherent in wounded skin, leading to bias [24]. In this study, a deep learning‐based framework is utilized to automate the segmentation of skin layers. The use of deep learning, specifically the U‐Net architecture, enables precise and objective identification of the skin layer. This automated approach enables precise and detailed calculation of wound‐tissue displacement and minimizes human error. To ensure the reliability and clinical relevance of the OCT‐derived stiffness metrics, this study includes a comparative assessment using the MyotonPRO, a commercially available, clinically established device for measuring soft‐tissue biomechanics. By comparing high‐resolution, localized OCT data with macroscopic measurements from MyotonPRO, this integrated framework provides a more robust validation of the observed biomechanical changes [25].

This study investigated the biomechanical properties of human skin, specifically focusing on variations in stiffness between the upper and lower limbs, and the therapeutic potential of LLLT in improving skin conditions during wound healing. While the individual techniques employed, including OCT, air‐jet indentation, and U‐Net‐based segmentation, are well‐established in their respective fields, this work presents an integrated, application‐oriented framework for quantifying skin stiffness in a clinical context. This system‐level integration enables a non‐contact assessment of the tissue's acute biomechanical response to LLLT by streamlining measurement. By leveraging a U‐Net‐based framework as an automated tool to segment skin layers, this study enables objective quantification of biomechanical stiffness from air‐jet indentation. The first hypothesis posits that skin stiffness varies across anatomical sites, reflecting local structural or functional adaptations rather than generalized differences between the upper and lower limbs. The second hypothesis suggests that stiffness measurements can serve as objective surrogate indicators of the immediate, short‐term biomechanical response of wound tissue to LLLT. By integrating these hypotheses, the research aims to explore the use of LLLT as a non‐invasive intervention for wound healing, with stiffness serving as an additional biomechanical marker to monitor immediate physiological response.

## 2 Materials and Methods

### 2.1 Participants

Participants were eligible if they were adult patients (≥ 18 years), presented with a chronic wound lasting more than 4 weeks, or had partial‐thickness burn wounds. Chronic and burn wounds were included based on delayed healing progression, consistent with prior studies demonstrating the beneficial effects of LLLT on chronic tissue injury [26]. However, participants were excluded if they were current smokers, as smoking impairs tissue oxygenation and microcirculation, potentially confounding the healing effects of LLLT [27]. Wounds that were fresh and still bleeding were also excluded to avoid complications, as active hemorrhage is a contraindication for laser therapy due to the possible vasodilation and risk of stimulating unwanted tissue response.

The studies involving human participants were approved by the Central Regional Research Ethics Committee of China Medical University, Taichung, Taiwan (CRREC‐112‐130) and subsequently registered in the International Trial Registry since 2025‐09‐16 [ClinicalTrials.gov: Identifier NCT07177274 (https://clinicaltrials.gov/study/NCT07177274)]. Before participating in the study, all participants were informed of its purpose and procedures. Written consent was obtained from all participants, who were assured that their information would be kept confidential. Following the Declaration of Helsinki, the respondents were allowed to withdraw from the study at any time, and their responses were anonymized. Participants must be at least 18 years old to be eligible.

All treatments were performed at Asia University Hospital, Taichung, Taiwan. The room temperature was maintained at 24°C ± 2°C. Temperature plays a crucial role in influencing the skin's properties. Research indicates that exposure to colder environments increases skin stiffness, which is attributed to decreased tissue elasticity. Conversely, warmer temperatures relax the skin and enhance its pliability [28].

### 2.2 Equipment

To evaluate the biomechanical response during wound healing, this study integrates several established techniques into a single assessment framework: OCT‐based air‐jet indentation with automated deep‐learning segmentation and the MyotonPRO device (Myoton AS, Tallinn, Estonia). By integrating structural (indentation‐based) and functional (impulse‐response) stiffness metrics, this study aims to provide a more nuanced, site‐specific understanding of tissue biomechanics following LLLT.

The OCT‐air‐jet indentation system enables non‐contact assessment of indentation stiffness by visualizing tissue deformation in response to controlled air pressure, providing high‐resolution tomographic insights into the short‐term biomechanical response, which serves as a surrogate imaging marker for tissue assessment. This study used an OCT device, OPXSV1‐02F (OPXION Tech. Incorporation, New Taipei City, Taiwan), to assess skin stiffness. The system provided a vertical optical resolution of 7.5 μm and a horizontal optical resolution of 10 μm, operating at 10 frames per second (fps) with an ROG ASUS laptop to install the OCT software (OPXION Imaging system V1.0) to obtain an image. The probe consisted of a 2 mm air‐pressure positive‐pressure port, a super‐luminescent diode light source operating at a central wavelength of 1310 nm, a nominal 350 nm dB spectral bandwidth, and an output power of 5 mW. The OCT system enabled imaging at depths of approximately 2 mm in highly scattering materials.

We proposed a non‐contact device design that uses air‐jet indentation and OCT to measure skin rigidity. Non‐contact OCT helps avoid aggravating wounded or sensitive skin, minimizing discomfort during measurement. Moreover, avoiding direct contact reduces the risk of introducing infections into compromised skin [29]. Suppose the OCT probe or air compressor device physically touched and pushed the skin. In this case, it may artificially alter the stiffness measurement because the skin is no longer in its natural resting state. To support the air‐jet indentation mechanism in OCT, we utilized a UNi‐CROWN 40 PSI 22 LPM Air Compressor (UNi‐CROWN Co. Ltd., New Taipei City, Taiwan) to generate a controlled air pulse that induced localized deformation on the skin surface. This deformation serves as a mechanical stimulus, enabling the dynamic displacement of skin layers, which is essential for evaluating skin stiffness.

**FIGURE 1:**
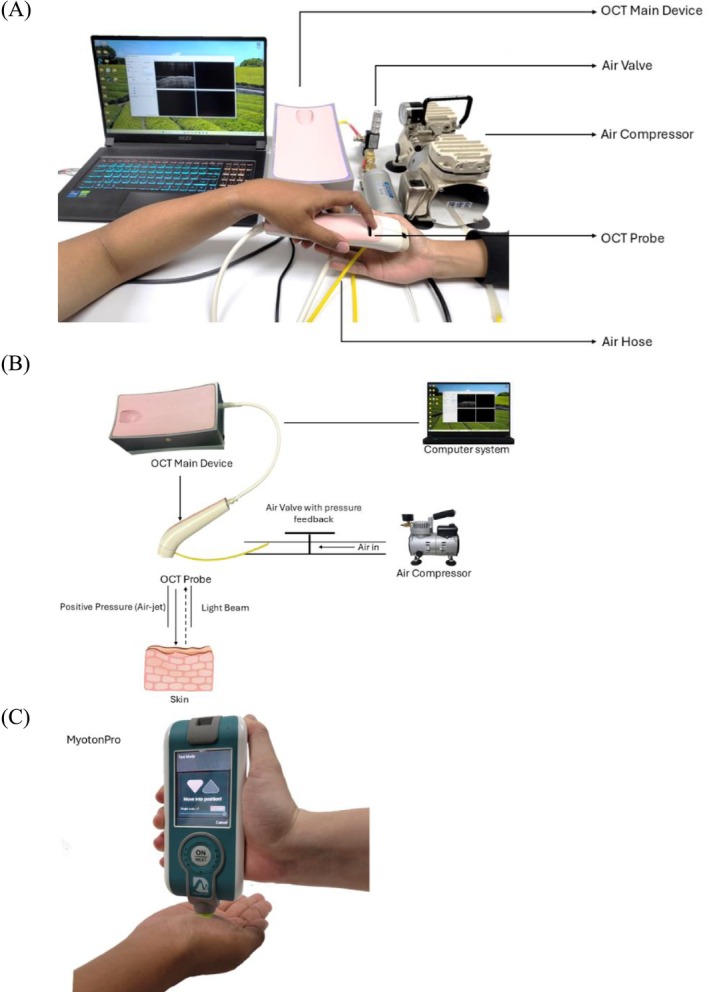
Protocols for measuring skin stiffness. (A) Illustration of the OCT air‐jet indentation. (B) OCT measurement of skin stiffness. (C) MyotonPRO measurement procedure. OCT, optical coherence tomography.

An electronic proportional valve with pressure feedback (ITV 1030‐311L‐Q, SMC Corporation, Tokyo, Japan), with a measuring range of 0.5 MPa, was installed between the air compressor and the OCT device. The OCT measurement procedure is illustrated in Figure 1A, which shows the setup and process for non‐invasive assessment of skin stiffness. Figure 1B conceptualizes the air‐jet indentation mechanism, highlighting its role in applying controlled pressure for stiffness evaluation.

In parallel, the MyotonPRO device served as a clinically established control measurement to validate the stiffness trends observed via OCT‐based air‐jet indentation. In this research, we use MyotonPRO (SN 000291, Myoton AS, Tallinn, Estonia), a non‐invasive, handheld digital device designed to assess the biomechanical properties of soft tissues. MyotonPRO delivers a brief mechanical impulse to the skin, inducing natural oscillations that are recorded to quantify parameters such as stiffness, elasticity, and tone. The measurement of stiffness using MyotonPRO is shown in Figure 1C.

### 2.3 Experimental Procedure

To ensure consistency and accuracy of the measurements, the OCT probe was positioned perpendicularly to the skin surface at a 90‐degree angle. The OCT probe was placed approximately 5 mm from the skin surface, maintaining a non‐contact distance. The participants were instructed to relax for 10 min to minimize the influence of recent physical activity or environmental stressors on their skin properties [30].

After the participants completed their rest period, stiffness data were measured on the opposite side of the wound to assess the normal skin. To minimize potential bias arising from differences between the dominant and non‐dominant sides, this measurement was used only as a reference baseline rather than as a directly matched control. This approach provided an internal comparison to evaluate the intervention's effects further. The OCT data collection process lasts 15 s. This included a 5 s before applying the air pressure to establish baseline conditions, 5 s while using the air pressure, and 5 s after turning off the air jet to allow the skin to return to its equilibrium state. This timeline ensured the reliability and accuracy of the collected data while minimizing potential interference from external factors. After the normal side, the wound stiffness before LLLT was measured using the OCT device.

**FIGURE 2:**
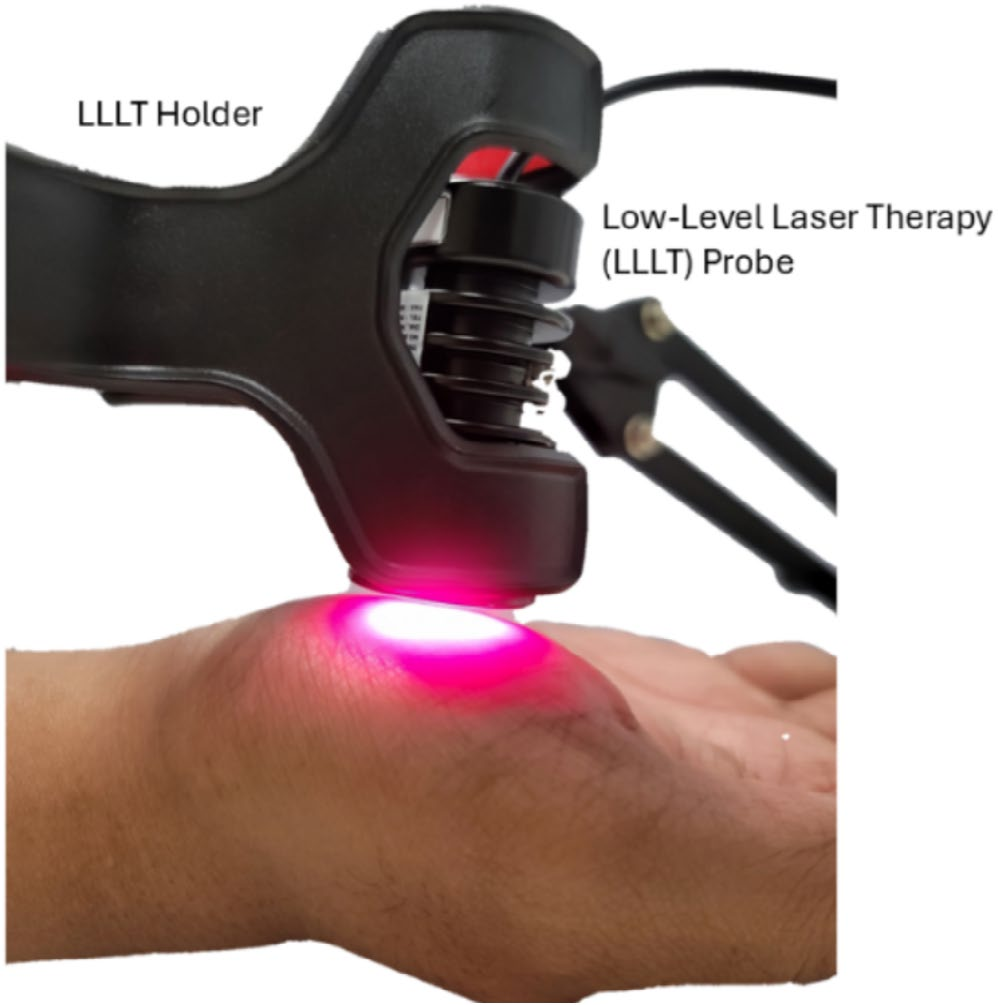
Application of the laser diode to the palm. LLLT, low‐level laser therapy.

After measuring wound stiffness with the OCT‐air‐jet indentation system, the next step was to apply LLLT to the wound for 3 min, then measure the wound stiffness again. Capture the tissue's acute, short‐term mechanical response. This study does not provide a longitudinal assessment of clinical healing over days or weeks; it focuses on immediate biomechanical shifts as surrogate indicators. The LLLT utilized a Red Dot Laser Diode Module Class IIIA (TIM201, 660 ± 10 nm, Output 80 ± 10 mW, Transverse Industries Co. Ltd., New Taipei, Taiwan). During the intervention procedure, the laser probe was positioned perpendicular to the skin surface at a 90‐degree angle, ensuring a non‐contact approach by maintaining a consistent distance of approximately 5 mm from the skin using a custom‐designed LLLT holder, which stabilized and secured the probe, as shown in Figure 2. Immediately following LLLT, the stiffness of the wound skin was assessed to capture its biomechanical condition under optimal post‐treatment circumstances.

### 2.4 Segmentation Procedure

To facilitate the quantification of the skin layer, a U‐Net‐based model was utilized as an automated segmentation tool. This established architecture was chosen due to its reliability in medical image segmentation. U‐Net is particularly suitable for medical imaging because it captures fine‐grained details and spatial hierarchies, making it ideal for segmenting layers such as the epidermis [31]. Ronneberger et al. [32] first introduced the U‐Net for biomedical image segmentation, demonstrating that a symmetric contracting–expanding path with skip connections can preserve fine spatial information essential for medical imaging tasks. Later, Shishkova et al. [33] successfully implemented a 3D U‐Net for human skin OCT images, achieving a dice coefficient of approximately 0.90 for the stratum corneum and 0.94 for deeper epidermal structures, underscoring the robustness of the U‐Net in delineating thin, low‐contrast skin layers. To ensure that the U‐Net approach was appropriate for OCT skin analysis, we first implemented the architecture on a smaller pilot dataset of 200 images for 100 training epochs. This preliminary validation yielded segmentation accuracies of 92.75% for the stratum corneum and 95.42% for the epidermis, confirming the method's reliability and suitability for subsequent large‐scale applications [34]. Based on these results, the current experiments were performed on a system equipped with an Intel(R) Core(TM) i7‐10 700 CPU and an NVIDIA GeForce RTX 3080 GPU. The dataset consisted of 700 images divided into three subsets: 60% for training, 20% for testing, and 20% for validation. To prevent data leakage and ensure generalization, the data were split at the patient level. This approach avoids the overlap of subject‐specific features across sets. This split ensures a balanced approach to model development, allowing effective learning, performance evaluation, and generalization. The U‐Net model was trained to segment the skin layers and predict biomechanical properties from these segmented areas. To quantify skin movement, the program automatically measured positional changes from the center of the segmented skin location before and after air pressure was applied. This enables accurate assessment of biomechanical responses, providing valuable insights into immediate mechanical behavior and short‐term tissue response.

The OCT image dataset underwent a pre‐processing step before being applied to the CNN model. Each image, originally obtained from 2D sequences of time‐bin files generated by the OCT device, was first converted to grayscale to simplify the data and reduce computational complexity while preserving essential structural information. The images were then uniformly cropped into a standardized 997 × 997 pixel square, corresponding to a physical size of 5 × 5 mm. To minimize random noise and enhance the image quality, a Gaussian filter was applied, which smoothed the images while retaining the critical features of the skin layers. Following this automated pre‐processing pipeline, manual masking was performed to isolate the Stratum Corneum (SC) region. This step was crucial because it directed the model's attention to the most relevant area for training, thereby reducing interference from background signals or other unrelated regions present in the OCT images. By focusing specifically on the segmented area, the dataset provided the model with more precise and meaningful input, which is particularly important in medical imaging tasks, where the accuracy of segmentation and subsequent analysis depends heavily on eliminating irrelevant or confounding features [35].

### 2.5 Data Analysis

**FIGURE 3:**
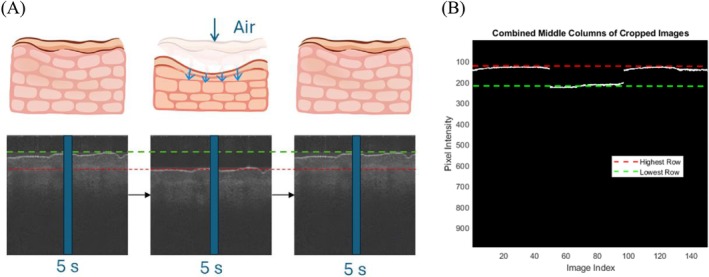
OCT skin deformation illustration. (A) Illustration of OCT skin deformation. (B) Combined middle columns of cropped images. OCT, optical coherence tomography; S, second.

Before applying air‐jet indentation, the initial skin depth was determined from each OCT image to establish a baseline for the subsequent deformation analysis. Once the air jet was activated, it induced a localized mechanical force on the skin, causing a measurable deformation. The resulting displacement was calculated as the difference between the skin's initial depth and its depth at the maximum indentation. To enhance the consistency and comparability of measurements across subjects, the central horizontal pixel line from each B‐scan was extracted to represent the tissue's axial intensity profile, as illustrated in Figure 3A. These centerline profiles from all images were then merged to generate a composite dataset, yielding an averaged representation of skin displacement behavior across the region of interest shown in Figure 3B. This approach enabled precise quantification of skin deformation in response to controlled air pressure, forming the basis for stiffness measurement. Mathematically, stiffness is expressed as Equation (1).

(1) $$K=\frac{F}{∆d}$$

The stiffness parameter (*K*) is defined as the ratio of the applied force (*F*) to skin displacement (Δ*d*). F represents the applied force, which is determined by multiplying the air‐jet pressure by the contact area, and ∆*d* denotes the force‐induced displacement. The stiffness parameter is useful for evaluating heterogeneous materials or layered structures, such as human skin, because it accounts for localized variations in mechanical properties and incorporates the sample's geometry.

U‐Net, a specialized convolutional neural network (CNN), is widely used in medical imaging for accurate image segmentation, particularly in areas requiring precise localization. The U‐Net architecture is well‐suited to medical images, such as OCT scans, which require detailed segmentation of skin layers to detect displacement caused by this force [36]. In this study, the U‐Net model was trained using 420 manually annotated OCT images to segment the SC layer. The model was then tested on 140 OCT images in the testing class to validate its performance by accurately segmenting the skin layers. All segmentation processes were conducted using MATLAB 2022b (MathWorks, Natick, MA, USA).

### 2.6 Statistical Analysis

The stiffness values are presented as mean ± standard error. A one‐way analysis of variance (ANOVA), followed by the least significant difference (LSD) post hoc test, was used to examine the effect of LLLT on skin stiffness across three statuses: normal skin, wound, and wound after therapy. Additionally, a student's *t*‐test was performed to compare wound skin stiffness between the upper and lower limbs. To assess the reliability and consistency of the stiffness measurements across repeated trials or raters, the intraclass correlation coefficient (ICC) was calculated. Given the modest sample size (*n* = 14) and potential variability in single measurements, results were interpreted based on average measures to ensure acceptable reliability, as recommended for clinical biomechanical data. To further evaluate the magnitude of the observed effects, Cohen's *d* effect sizes were calculated along with 95% confidence intervals (95% CI) to provide an accurate measure of precision and improve the interpretability of the results. Pearson's correlation analysis was conducted to examine the relationship between skin stiffness values obtained from OCT‐based air jet indentation and those measured using the MyotonPRO device. This analysis aimed to assess the degree of association between these two modalities. All statistical analyses were performed using SPSS version 22 (IBM, NY, USA), with a significance level set at *p* < 0.05.

## 3 Results

### 3.1 Segmentation Result

**FIGURE 4:**
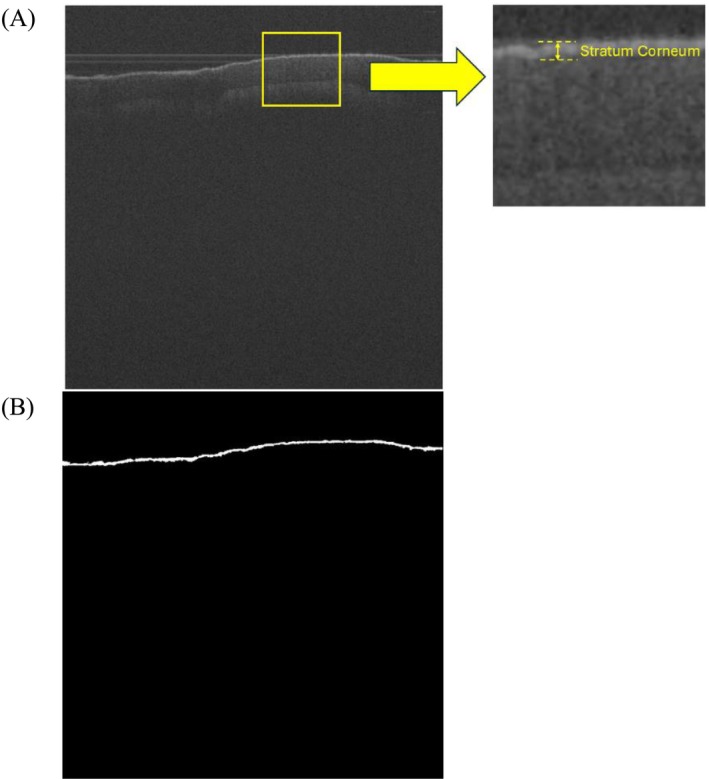
Illustration of skin images measured using OCT. (A) Original image; (B) segmented SC skin layer image. OCT, optical coherence tomography; SC, stratum corneum.

The study included 14 participants with wounds (6 men and 8 women) aged 19–77 years, with a mean (±SD) age of 33.6 ± 15.8 years, body weight of 57.6 ± 12.8 kg, height of 162.3 ± 8.3 cm, and body mass index (BMI) of 35.4 ± 7.5 kg/m^2^. The U‐Net model effectively automated the segmentation of the skin layers, providing a consistent basis for subsequent stiffness analysis. The accuracy for segmenting the stratum corneum (SC) reached 92%, demonstrating a significant improvement compared to traditional methods. A dice coefficient of 0.89 and an Intersection over Union (IoU) of 0.82 further confirmed the model's robustness. Additionally, the loss function converged efficiently, stabilizing at 0.045 after 50 epochs, highlighting the model's strong generalization capabilities. These results indicate that U‐Net successfully captures fine details, making it an optimal choice for this study. Skin layer segmentation is shown in Figure 4.

### 3.2 Effect of LLLT on Different Locations

**FIGURE 5:**
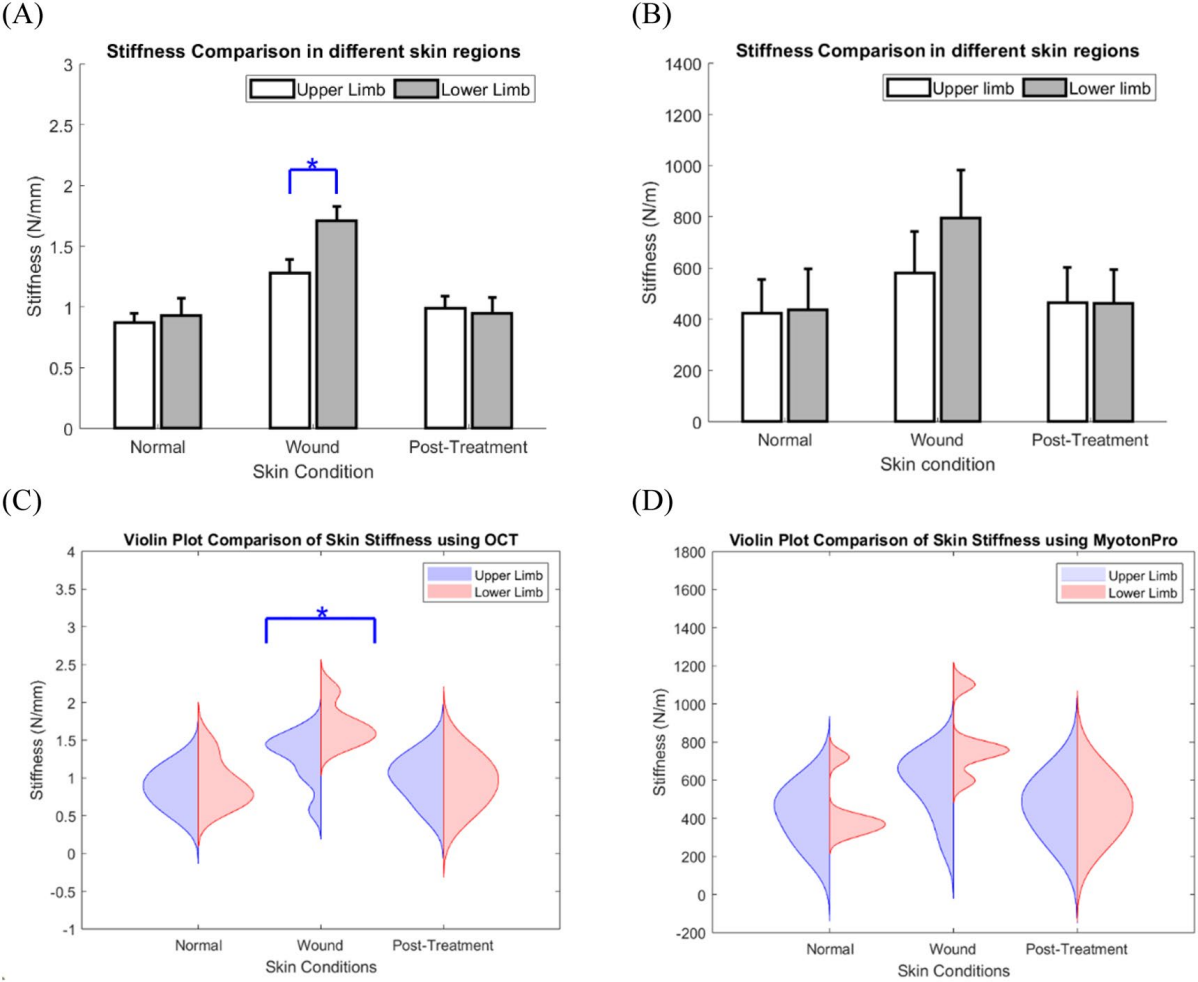
Chart comparison (A) Bar chart comparisons of skin stiffness at different locations measured by OCT‐based air jet indentation; (B) bar chart comparisons of skin stiffness at different locations measured by MyotonPRO; (C) violin plot comparisons of skin stiffness at different locations measured by OCT‐based air jet indentation; (D) violin plot comparisons of skin stiffness at different locations measured by MyotonPRO. Bar chart data were shown as mean ± standard errors. m, meter; mm, millimeter; N, newton; OCT, optical coherence tomography; *, significant difference (*p* < 0.05).

Additionally, the Student's *t*‐test for skin stiffness was measured using OCT‐based air jet indentation, revealing significant differences in the wound skin. The upper limb was significantly lower than that of the lower limb (1.28 ± 0.11 vs. 1.71 ± 0.12, *p* = 0.03), as shown in (Table 1, Figure 5A,C). However, there was no significant difference in the student's *t*‐test for skin stiffness measured using MyotonPRO, as shown in (Table 1, Figure 5B,D).

**TABLE 1:** Comparison of stiffness at different skin locations.

Device	Skin status	Location		Student's *t*‐test
		Upper limb (mean ± SE)	Lower limb (mean ± SE)	*p*
OCT	Normal (N/mm)	0.87 ± 0.08	0.93 ± 0.14	0.72
	Wound (N/mm)	1.28 ± 0.11	1.71 ± 0.12	0.03*
	After treatment (N/mm)	0.99 ± 0.10	0.95 ± 0.13	0.80
MyotonPRO	Normal (N/m)	422.96 ± 43.76	435.54 ± 72.62	0.89
	Wound (N/m)	579.52 ± 54.24	794.46 ± 83.47	0.07
	After treatment (N/m)	463.40 ± 46.15	461.40 ± 58.77	0.98

*Note:* Data are presented as mean ± standard error. *, significant difference (*p* < 0.05).

Abbreviations: m, meter; mm, millimeter; N, newton; OCT, optical coherence tomography.

### 3.3 Effect of LLLT on Different Statuses

**FIGURE 6:**
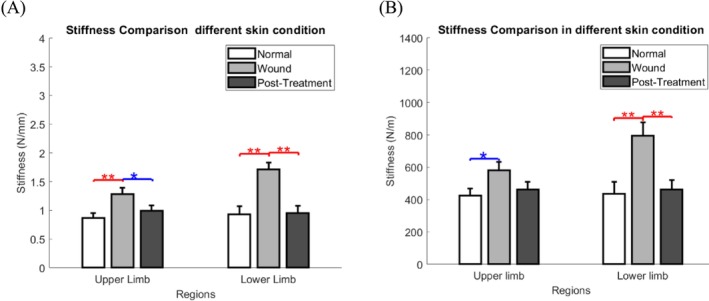
Chart comparison (A) Bar chart comparisons of skin stiffness under different conditions measured by OCT‐based air jet indentation; (B) bar chart comparisons of skin stiffness under different conditions measured by MyotonPRO. Data are shown as mean ± standard error. m, meter; mm, millimeter; N, newton; OCT, optical coherence tomography; *, significant difference (*p* < 0.05); **, significant difference (*p* < 0.01).

The effect of LLLT was analyzed using one‐way analysis of variance (ANOVA), which showed that the impact of LLLT differed significantly between wound skin and after treatment with OCT, in both the upper and lower limbs (Table 2 and Figure 6A). The results demonstrated that normal skin had significantly higher stiffness than wound skin in the upper (0.87 ± 0.08 vs. 1.28 ± 0.11 N/mm, *p* = 0.006) and lower (0.93 ± 0.14 vs. 1.71 ± 0.12 N/mm, *p* = 0.001) limbs. In addition, the wound skin after treatment was significantly lower than that in the upper (1.28 ± 0.11 vs. 0.99 ± 0.10 N/mm, *p* = 0.047) and lower (1.71 ± 0.12 vs. 0.95 ± 0.13 N/mm, *p* = 0.002) limbs.

**TABLE 2:** Comparison of stiffness in different skin conditions.

Location	Skin status			One‐way ANOVA	LSD		
					Post hoc		
	Normal (mean ± SE)	Wound (mean ± SE)	After treatment (mean ± SE)	*p*	Normal versus wound	Normal versus after treatment	Wound versus after treatment
OCT upper limb (N/mm)	0.87 ± 0.08	1.28 ± 0.11	0.99 ± 0.10	0.018*	0.006**	0.375	0.047*
OCT lower limb (N/mm)	0.93 ± 0.14	1.71 ± 0.12	0.95 ± 0.13	0.001**	0.001**	0.907	0.002**
MyotonPRO upper limb (N/m)	422.96 ± 43.76	579.52 ± 54.24	463.4 ± 46.15	0.911	0.031*	0.559	0.102
MyotonPRO lower limb (N/m)	435.54 ± 72.62	794.46 ± 83.47	461.4 ± 58.77	0.007**	0.004**	0.805	0.007**

*Note:* Data are presented as mean ± standard error. *, significant difference (*p* < 0.05); **, significant difference (*p* < 0.01).

Abbreviations: m, meter; mm, millimeter; N, newton; OCT, optical coherence tomography.

When using MyotonPRO, the stiffness of normal skin was significantly lower than that of wound skin in the upper (422.96 ± 43.76 vs. 579.52 ± 54.24 N/m, *p* = 0.031) and lower (435.54 ± 72.62 vs. 794.46 ± 83.47 N/m, *p* = 0.04) limbs. However, the wound skin stiffness was significantly higher in the wound skin after therapy only in the lower limb (794.46 ± 83.47 vs. 461.4 ± 58.77 N/m, *p* = 0.007). In the upper limb, MyotonPRO did not detect a statistically significant change post‐treatment, suggesting that the OCT‐based system may be more sensitive to acute, subtle biomechanical modulations in more compliant tissue areas, as shown in (Table 2 and Figure 6B).

An ICC analysis was conducted using a two‐way mixed‐effects model to evaluate the reliability of the stiffness measurements across the six experimental conditions (three skin statuses × two anatomical locations). The ICC for single measures was 0.254, indicating poor reliability when considering the individual stiffness measurements. However, the ICC for average measures was 0.671, reflecting moderate reliability when measurements were averaged across conditions. Consequently, all subsequent biomechanical assessments in this study were based on average values to ensure a more stable and representative estimate of skin stiffness. Table 3 summarizes the ICC for skin stiffness, including the 95% CI.

**TABLE 3:** Intraclass correlation coefficient of skin stiffness.

Measurements	Intraclass correlation	95% CI
single measures	0.254	0.039–0.771
Average measures	0.671	0.164–0.953

Abbreviation: CI, confidence interval.

Cohen's *d* analysis revealed that using OCT in the upper limb, normal skin compared to wound skin showed a strong effect (*d* = 1.33). Compared with after‐treatment skin, normal skin showed a moderate effect (*d* = 0.55). Compared to the after‐treatment skin, the wound skin showed a moderate effect (*d* = 0.75). In the lower limbs, normal skin compared to wound skin showed a strong effect (*d* = 2.71). Compared to the after‐treatment skin, normal skin showed a weak effect (*d* = 0.08). Compared to the after‐treatment skin, the wound skin showed a strong effect (*d* = 2.57). In addition, using MyotonPRO showed similar results; in the upper limb, normal skin compared to wound skin showed a strong effect (*d* = 1.41). Compared with after‐treatment skin, normal skin showed a modest effect (*d* = 0.46). Compared to the after‐treatment skin, the wound skin showed a moderate effect (*d* = 0.93). In the lower limbs, normal skin compared to wound skin showed a strong effect (*d* = 2.65). Compared to the after‐treatment skin, normal skin showed a weak effect (*d* = 0.08). Compared to the after‐treatment skin, the wound skin showed a strong effect (*d* = 2.64). Table 4 summarizes the effect size of skin stiffness in different statuses, including the mean differences and 95% CI for the mean differences.

**TABLE 4:** Effect size of skin stiffness in different statuses.

Device	Location	Skin status	Mean difference	Effect size (Cohen's *d*)	95% CI
OCT (N/mm)	Upper limb	Normal skin versus wound skin	−0.42	1.33	−0.70 to −0.13
		Normal skin versus after treatment	−0.13	0.55	−0.41 to 0.16
		Wound skin versus after treatment	0.29	0.75	0.01 to 0.58
	Lower limb	Normal skin versus wound skin	−0.78	2.71	−1.18 to −0.37
		Normal skin versus after treatment	−0.02	0.08	−0.43 to 0.38
		Wound skin versus after treatment	0.75	2.57	0.35 to 1.16
MyotonPRO (N/m)	Upper limb	Normal skin versus wound skin	−156.57	1.41	−297.44 to −15.77
		Normal skin versus after treatment	−40.44	0.46	−181.30 to 100.41
		Wound skin versus after treatment	116.12	0.93	−24.73 to 256.98
	Lower limb	Normal skin versus wound skin	−358.92	2.65	−581.80 to −136.05
		Normal skin versus after treatment	−25.86	0.08	−248.73 to 197.01
		Wound skin versus after treatment	333.06	2.64	110.19 to 555.93

Abbreviations: CI, confidence interval; m, meter; mm, millimeter; N, newton; OCT, optical coherence tomography.

Additionally, based on OCT, the analysis revealed that comparisons of the upper and lower limbs in normal skin showed a modest effect (*d* = 0.23), in wound skin showed a strong effect (*d* = 1.33), and in after‐treatment skin showed a weak effect (*d* = 0.14). However, using MyotonPRO, the analysis revealed that comparisons of upper and lower limbs in normal skin showed a modest effect (*d* = 0.22), in wounded skin showed a strong effect (*d* = 1.37), and in after‐treatment skin showed a weak effect (*d* = 0.14). Table 5 summarizes the effect size of skin stiffness in different statuses, including the mean differences and 95% CI for the mean differences.

**TABLE 5:** Effect size of skin stiffness in different upper and lower limb locations.

Device	Skin status	Mean difference	Effect size (Cohen's *d*)	95% CI
OCT (N/mm)	Normal skin	−0.06	0.23	−0.39 to 0.27
	Wound skin	−0.42	1.33	−0.81 to −0.04
	After treatment skin	0.04	0.14	−0.31 to 0.39
MyotonPRO (N/m)	Normal skin	−12.58	0.09	−213.23 to 188.06
	Wound skin	−214.94	1.23	−447.60 to 17.73
	After treatment skin	2.00	−0.02	−167.67 to 171.67

Abbreviations: CI, confidence interval; m, meter; mm, millimeter; N, newton; OCT, optical coherence tomography.

### 3.4 Correlation Result

**FIGURE 7:**
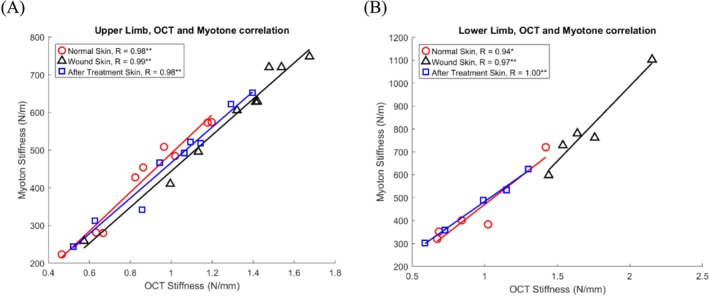
Correlation between skin stiffness measured using OCT‐based air jet indentation and MyotonPRO in three different skin conditions (normal, wound, and after treatment skin). (A) Correlation in upper limbs. (B) Correlation in the lower limbs. m, meter; mm, millimeter; N, Newton; OCT, optical coherence tomography. *, significant correlation (*p* < 0.05); **, significant correlation (*p* < 0.01).

Skin stiffness measurements across all physiological conditions (normal, wound, and after treatment) in the upper and lower limb locations exhibited a consistent positive correlation when assessed using both OCT‐based air jet indentation and MyotonPRO devices (Figure 7A,B and Table 6). This correlation suggests that, despite localized variations due to injury or therapeutic intervention, the biomechanical properties of the skin maintain a degree of systemic coherence across anatomical locations. The convergence of results from two distinct modalities, OCT‐based air jet indentation and handheld MyotonPRO, further reinforces the general trend and validity of the stiffness metrics as surrogate indicators of tissue response. These findings offer valuable insights into clinical monitoring and therapeutic evaluation.

**TABLE 6:** Correlation coefficients in different locations based on OCT and MyotonPRO at three different skin statuses.

Locations	Skin status	*R*	*p*
Upper limb	Normal skin	0.98	0.00**
	Wound skin	0.99	0.00**
	After treatment skin	0.98	0.00**
Lower limb	Normal skin	0.94	0.02*
	Wound skin	0.97	0.01**
	After treatment skin	0.99	0.00**

*Note:* Data are shown as correlation coefficients; *, significant correlation (*p* < 0.05); **, significant correlation (*p* < 0.01).

## 4 Discussion

By integrating a U‐Net‐based architecture for automated segmentation and an air‐jet indentation system for stiffness quantification, this study presents a functional system‐level framework for assessing skin tissue response. This integrated approach observed that LLLT for 3 min significantly reduced skin stiffness in wounded areas, although the stiffness remained higher than that of normal skin. These findings suggest that LLLT contributes to structural modifications in the skin, thereby modulating its mechanical properties. OCT measurements revealed significant differences in skin stiffness after LLLT in both upper and lower limbs. In contrast, MyotonPRO analysis only detected a significant difference in the lower limbs, whereas OCT detected changes in both the upper and lower limbs. This suggests that OCT may offer greater sensitivity in detecting acute biomechanical shifts across different anatomical regions immediately following intervention.

The utility of the standard U‐Net architecture for segmenting the SC layer stems from its established symmetrical encoder‐decoder structure, which combines feature extraction and precise localization. This setup, enhanced by skip connections, ensures that critical spatial details are maintained throughout the network, making it particularly advantageous for analyzing thin and morphologically intricate skin layers in OCT images [32]. The model's ability to fuse detailed texture patterns with broader contextual information is essential for reliably outlining the SC [37]. In this study, the application of this established model achieved high accuracy, aligning with previous findings [33]. These results confirm that leveraging existing deep learning tools provides reliable boundary detection, which is essential for the subsequent biomechanical analysis within this integrated framework.

OCT‐based air jet indentation assessed skin stiffness and revealed significant differences between normal and wounded skin in the patients' upper and lower limbs. This non‐contact method enables precise measurement of the mechanical properties of tissues, making it ideal for evaluating variations due to injury. Injured skin showed increased stiffness, mainly due to alterations in the extracellular matrix (ECM), particularly involving excessive collagen deposition and fiber cross‐linking. This is part of the normal wound healing process, which includes fibroblast activation and ECM remodeling to restore tissue integrity [38]. Excessive collagen buildup, especially when disorganized, is associated with keloid and hypertrophic scar formation, where fibroblasts produce abnormal amounts of collagen in response to extrinsic tensile forces and altered cellular mechanics [39]. This leads to reduced skin flexibility and increased tissue stiffness.

**FIGURE 8:**
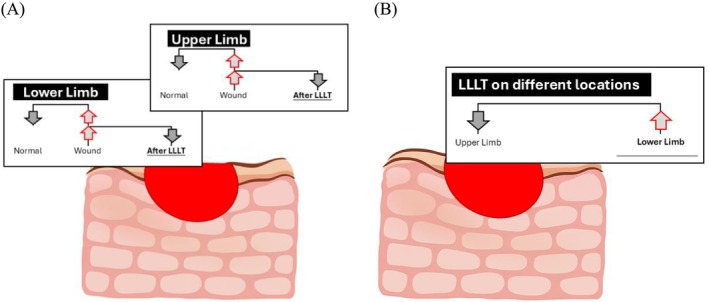
Illustration of the effect of LLLT at different locations and under different conditions. (A) The effect of LLLT showed that skin stiffness in the wound on the lower limb was higher than that on the upper limb. (B) The effect of LLLT in different skin conditions showed that skin stiffness in wounds on the upper and lower limbs was higher than normal, and after treatment. LLLT, low‐level laser therapy.

Following a 3 min LLLT session, a significant reduction in skin stiffness was observed at both limb locations, as illustrated in Figure 8A. LLLT exerts its effects by absorbing red light at 660 nm by mitochondrial chromophores, enhancing ATP production, and modulating reactive oxygen species. These changes promote gene expression related to tissue repair, stimulate fibroblast proliferation, and enhance collagen remodeling, thereby improving skin elasticity [11]. Importantly, LLLT facilitates the transition from type III to type I collagen, improves collagen fiber alignment, and increases dermal hydration, all of which contribute to decreased stiffness, which is associated with the immediate modulation of the wound's mechanical environment [40]. The significance of utilizing stiffness as a surrogate marker is reinforced by its strong correlation with these established biological indicators of wound healing. Previous studies have demonstrated that a reduction in tissue stiffness often parallels the transition from disorganized granulation tissue to a more structured extracellular matrix, characterized by the maturation of collagen fibers and a shift from type III to type I collagen [41].

Moreover, LLLT enhances microcirculation and cellular metabolism, improving oxygen and nutrient delivery to the wound site, further supporting tissue remodeling where linked to mechanical softening [9]. It also enhances fibroblast function and promotes better wound contraction, leading to measurable softening of previously stiff skin [42]. Although post‐treatment stiffness remains higher than normal skin, these effects demonstrate LLLT's potential to induce immediate, short‐term biomechanical shifts in the wounded tissue. By observing these acute biomechanical shifts, our study provides an early‐stage indicator that aligns with structural and circulatory improvements typically assessed using more invasive histological or biochemical methods.

Additionally, the MyotonPRO result, which assesses the biomechanical properties of soft tissues through brief mechanical impulses, revealed a significant reduction in skin stiffness in wounded skin of the lower limbs after LLLT. In contrast, the upper limbs did not exhibit such changes. This contrast may arise from the fundamental differences in how OCT‐based air jet indentation and MyotonPRO assess tissue stiffness. OCT‐based air jet indentation is a high‐resolution, non‐contact technique that provides localized stiffness measurements at microscopic depths, particularly in the epidermis and superficial dermis, which are typically the first layers affected by LLLT‐induced remodeling [11]. In contrast, MyotonPRO measures the mechanical response of soft tissue over a larger, more integrated area that includes deeper structures, such as the subcutaneous tissue and muscle, and is influenced by overlying and underlying tissue layers [43].

Consequently, subtle changes in the superficial layers of the skin, such as those often observed in the upper limbs following short LLLT sessions, may be too shallow or localized to impact the overall mechanical response measured by MyotonPRO significantly. Furthermore, the MyotonPRO impulse may not generate sufficient resolution to detect microstructural softening unless widespread changes involve the deeper connective tissue [44]. Conversely, OCT‐based air jet indentation provides a sensitive measure of mechanical changes within a confined focal volume, detecting even minor variations in tissue biomechanics that may not significantly influence whole‐tissue mechanical behavior [45]. This explains why stiffness reduction in the upper limb after LLLT was captured by OCT but not by MyotonPRO, which is more responsive to macroscopic or high‐magnitude biomechanical alterations. Hence, integrating these two established assessment tools provides a comprehensive framework. The use of MyotonPRO as a clinically established benchmark validates the significance of the localized, high‐resolution surrogate markers captured by OCT, ensuring that the observed shifts reflect real mechanical alterations [46].

Wounds in the lower limbs often exhibit higher stiffness than those in the upper limbs, primarily because of the unique physiological and biomechanical characteristics of the lower extremities, as illustrated in Figure 8B. The lower limbs bear the body's weight and are subject to constant mechanical stress, which influences tissue remodeling and stiffness during wound healing [47]. Additionally, the lower limbs are more prone to circulatory issues, such as chronic venous insufficiency, which can impair wound healing and increase tissue stiffness [48]. These factors combined can lead to a more fibrotic healing response, resulting in stiffer scar tissue in the lower limbs than in the upper limbs. Biochemical changes after LLLT improve cellular energy availability and promote vasodilation, thereby increasing oxygen and nutrient delivery to the wound site. This is especially beneficial in the lower limbs, where vascular perfusion is often compromised by chronic venous insufficiency or gravitational pressure.

The results of this study align well with the expected behavior of skin stiffness at different stages of wound healing and LLLT intervention. Only comparisons of the upper and lower limbs in the wound skin showed a significant difference. This reinforces the idea that wounds in the lower limbs are often more severe or take longer to heal initially, a common clinical observation [49]. Importantly, this significant difference highlights the baseline imbalance in wound conditions prior to any therapeutic intervention, as reported in several wound healing studies [22]. After applying LLLT, the absence of a significant difference between the limbs suggests that the therapy helped to normalize skin stiffness across locations. This suggests that LLLT may temporarily mitigate regional disparities in tissue stiffness, potentially aligning the immediate mechanical state of wounds in both the upper and lower limbs. Furthermore, the lack of a significant difference in normal skin between the two locations confirms that any observed differences in the wound condition are not due to inherent anatomical differences, but rather the presence and severity of the wound itself [50]. Together, these findings suggest that LLLT may play a modulatory role in addressing regional disparities in skin stiffness, serving as an acute indicator of treatment response, especially in the lower limbs [51]. The novelty of this work lies in the system‐level integration of established biophotonic and computational methods, rather than in the development of new underlying algorithms. This integrated, application‐oriented framework serves as a practical tool for monitoring acute tissue responses, providing a bridge between high‐resolution optical imaging and clinical biomechanical assessment.

Despite these promising findings, this study had two limitations. First, this study assessed wound stiffness at only one time point, which may not capture the temporal effects of the therapy. LLLT outcomes can vary, and a single measurement may overlook these dynamics. For instance, a study comparing LLLT and sham treatments found significant differences in pain pressure thresholds at the 8‐week mark, suggesting that multiple assessments over time are essential for understanding the full impact of the therapy [52]. Second, the collection of stiffness data immediately after LLLT may not account for delayed therapeutic effects. Some benefits of LLLT, such as enhanced wound contraction, may become evident only after a specific period. In a previous study by Hopkins [53], LLLT‐treated lesions showed 153% greater contraction at day 6, followed by 55% at day 8, and 22% at day 10. Differences were minimal before day 6, indicating a delayed effect on contraction depending on the wound type. Third, the disparity between single‐measure and average‐measure ICCs warrants careful consideration. The lower reliability in single trials likely stems from the dynamic nature of wounded tissue and the OCT‐air‐jet indentation's high sensitivity to minor participant movements. However, the moderate reliability achieved with averaged measures (ICC = 0.671) aligns with previous practices in clinical biomechanics [54, 55]. This confirms that while point‐specific measurements may vary, the overall integrated framework provides a consistent trend for monitoring the acute response to LLLT. Fourth, the study included a wide age range of participants (19–77 years) with a modest sample size, which may introduce variability in baseline skin elasticity due to natural aging processes. To mitigate this factor, the analysis focused on relative biomechanical changes, ensuring that the observed effects specifically reflect the immediate response to LLLT [56].

## 5 Conclusion

The OCT–air‐jet indentation system, combined with deep learning, successfully demonstrated quantifiable differences in skin stiffness across anatomical sites, supporting our first hypothesis. In addition, the system captured immediate biomechanical shifts in the skin following LLLT application, suggesting that stiffness measurements can serve as objective surrogate markers to monitor the short‐term mechanical response of wound tissue. Integrating a deep learning‐based U‐Net architecture enabled precise segmentation of the skin layers, enhancing the accuracy and consistency of the stiffness measurements, with a segmentation accuracy of 92%. The results revealed that wound skin stiffness in the lower limb was significantly higher than that in the upper limb. The significant reduction in skin stiffness observed 3 min post‐LLLT indicates an immediate modulation of the tissue's mechanical properties, providing an objective measure of the short‐term response to the therapy. The lower limb had higher skin stiffness at the wound site. Our results also indicate that LLLT acutely modulates wound stiffness towards values more closely resembling those of non‐injured tissue. However, the measurements reflect an immediate biomechanical shift rather than definitive clinical recovery. This study demonstrated immediate changes in skin stiffness following LLLT that may serve as surrogate indicators of the treatment's impact on tissue mechanics, particularly in wounds located on the lower limbs. The significant reduction in stiffness observed immediately post‐treatment reflects a short‐term biomechanical response rather than a longitudinal assessment of definitive wound‐healing efficacy. Future long‐term studies are required to correlate these acute mechanical shifts with clinical healing outcomes.

## Author Contributions

Conceptualization: Y.‐K.J. and C.‐W.L. Methodology: G.T.R., H.‐T.C., and W.‐C.S. Supervision: B.‐Y.L., C.‐C.T., S.C.P., K.V., W.C.‐C.L., and C.T.S.C. Writing‐review and editing: Y.‐K.J. and C.‐W.L. Writing‐original draft: G.T.R. Investigation: B.‐Y.L., C.‐C.T., S.C.P., K.V., W.C.‐C.L., and C.T.S.C. All authors have read and agreed to the published version of this manuscript.

## Funding

This study was supported by grants from the National Science and Technology Council of Taiwan (NSTC 114‐2923‐E‐468‐001‐MY3 and NSTC 114‐2410‐H‐468‐016). The funding agency was not involved in the data collection, analysis, or interpretation.

## Ethics Statement

The studies involving humans were approved by Central Regional Research Ethics Committee China Medical University, Taichung, Taiwan (CRREC‐112‐130). This study was registered in the International Trial Registry [ClinicalTrials.gov: Identifier NCT07177274] with the first posted of Study Registration Dates on 2025‐09‐16. The study was conducted in accordance with the local legislation and institutional requirements.

## Consent

All participants provided written informed consent to participate in this study.

## Conflicts of Interest

The authors declare no conflicts of interest.

## Data Availability

The authors confirm that the data supporting the findings of this study are available in this article.
